# Predisposing Factors, Pathologies, and Precipitating Factors Causing Intracerebral Hemorrhage

**DOI:** 10.1161/STROKEAHA.125.051831

**Published:** 2025-12-03

**Authors:** Alice Hosking, Neshika Samarasekera, Tom J. Moullaali, William N. Whiteley, Vega Pratiwi Putri, Mark A. Rodrigues, Colin Smith, Santosh B. Murthy, David Gaist, Paula Muñoz Venturelli, Xin Cheng, Craig S. Anderson, Ashkan Shoamanesh, Rustam Al-Shahi Salman

**Affiliations:** Centre for Clinical Brain Sciences (A.H., N.S., T.J.M., W.N.W., V.P.P., M.A.R., C.S., R.A.-S.S.), The University of Edinburgh, United Kingdom.; Edinburgh Clinical Trials Unit, The Usher Institute (R.A.-S.S.), The University of Edinburgh, United Kingdom.; British Heart Foundation Data Science Centre, Health Data Research, London, United Kingdom (W.N.W.).; Neurology Department, Universitas Gadjah Mada, Yogyakarta, Indonesia (V.P.P.).; Clinical and Translational Neuroscience Unit, Feil Family Brain and Mind Research Institute and Department of Neurology, Weill Cornell Medicine, New York, NY (S.B.M.).; Research Unit for Neurology, Odense University Hospital, University of Southern Denmark (D.G.).; Centro de Estudios Clínicos, Instituto de Ciencias e Innovación en Medicina, Facultad de Medicina Clínica Alemana Universidad del Desarrollo, Santiago, Chile (P.M.V.).; Department of Neurology, National Center for Neurological Disorders, National Clinical Research Centre for Aging and Medicine, Huashan Hospital (X.C.), Fudan University, Shanghai, China.; The Institute of Science and Technology for Brain-Inspired Intelligence (C.S.A.), Fudan University, Shanghai, China.; The George Institute for Global Health, University of New South Wales, Camperdown, Australia (C.S.A.).; Department of Medicine, Population Health Research Institute, McMaster University, Hamilton, ON, Canada (A.S.).

**Keywords:** cerebral hemorrhage, cerebral small vessel diseases, hypertension, risk factors, stroke

## Abstract

Most people with spontaneous intracerebral hemorrhage (ICH) have hypertension, which is the strongest modifiable predisposing (risk) factor. However, multiple long-term medical conditions and other known predisposing factors for ICH usually coexist with hypertension, indicating that the causal pathway is multifactorial, and the term hypertensive ICH is oversimplistic. In this review, we integrate the highest quality evidence and our clinical experience in a framework to attribute multiple predisposing factors, underlying pathologies, and precipitating factors as the cause of ICH. In clinical practice, this framework supports physicians to take a holistic approach to treatment and prevention of ICH. In research, this framework shows how existing classification systems for the cause of ICH include underlying macrovascular, microvascular, and other structural pathologies but few predisposing or precipitating factors. Furthermore, this framework can inform the development of a more holistic classification system and expose knowledge gaps, including how predisposing factors lead to underlying pathologies and why only some people with these pathologies experience ICH.

Acute spontaneous (nontraumatic) intracerebral hemorrhage (ICH) causes one-third of strokes and half the deaths and disability due to stroke worldwide.^1^ The crude incidence of ICH increased by 43% worldwide between 1990 and 2019, and rates in lower- and middle-income countries are 2× to 4× greater than in high-income countries. Effective prevention strategies and treatments are essential for reducing the burden of this devastating disease.

Specific genetic, environmental, or physiological predisposing (risk) factors may lead to the pathologies that underlie ICH. Prospective cohorts have demonstrated that 15% of ICHs have an underlying macrovascular abnormality, and most of the remainder are attributable to cerebral small vessel diseases (cSVDs) such as arteriolosclerosis, cerebral amyloid angiopathy (CAA), a combination of the two, or rarer sporadic and genetic subtypes.^2^ Although high blood pressure (BP) is the strongest modifiable predisposing factor for ICH,^3^ most people with high BP do not develop ICH, suggesting that other factors contribute. The largest case-control study to date found that people with ICH commonly have multiple predisposing factors, including alcohol misuse, high waist-to-hip ratio, reduced physical activity, poor diet, psychosocial factors, and cardiac causes.^4^

In this review, we start by summarizing the highest quality evidence about the many predisposing factors, underlying pathologies, and precipitating factors for spontaneous ICH. Next, we describe a holistic approach that physicians can take to recognize and address the many factors contributing to the cause of spontaneous ICH. Finally, we critically appraise how published causal classification systems for ICH have attempted to simplify the complexity of causation and conclude by identifying knowledge gaps and the next steps for research to develop a more inclusive causal classification system for ICH.

## Predisposing Factors for Spontaneous ICH

Predisposing factors determine the development of pathologies that lead to ICH. Although these predisposing factors likely interact, most studies have considered them individually. We conducted a search for systematic reviews and large cohort and case-control studies of predisposing factors for ICH (Supplemental Material), found over 30 putative protective or predisposing factors (Table S1), and listed the predisposing factors with consistent clinical evidence or strong mechanistic support for their association with spontaneous ICH in our framework (Figure).

**Figure.**
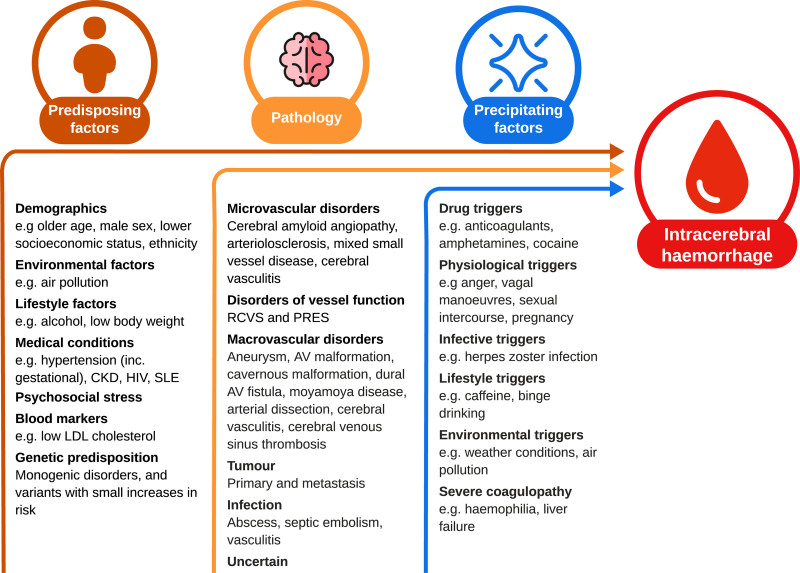
**Predisposing factors, pathologies, and precipitating factors causing intracerebral hemorrhage.** Examples were chosen based on biological plausibility, reliability, consistency, and strength of available evidence (Supplemental Material). AV indicates arteriovenous; CKD, chronic kidney disease; LDL, low-density lipoprotein; PRES, posterior reversible encephalopathy syndrome; RCVS, reversible cerebral vasoconstriction syndrome; and SLE, systemic lupus erythematosis.

### Demographic Predisposing Factors

In common with other cardiovascular diseases, spontaneous ICH is associated with aging, male sex, and social deprivation (Table S1).^1,5^ A combination of genetic, economic, and cultural factors may explain the association with East and South Asian ethnicity.^6^ In the United States, people of the Black race and Hispanic ethnicity have higher age-standardized ICH incidence rates.^7^ Later age of menopause is a sex-specific predisposing factor.^8^

### Genetic Predisposition

Genetic traits may determine some predisposing factors or pathologies underlying ICH. Most knowledge about these traits is derived from populations in Europe and North America, with little information from populations at highest risk in sub-Saharan Africa and South-East Asia. Few epigenetic studies have explored potential interactions between other (eg, environmental) predisposing factors and genetic traits.

Rare monogenic disorders predispose to pathologies that confer a higher risk of ICH, such as CAA via point mutations in the amyloid precursor protein or cystatin C^9^ and familial cerebral cavernous malformation via mutations in *KRIT1*, *CCM2*, or *PDCD10*.^10^ People with Down syndrome have 3 copies of the amyloid precursor protein gene and high rates of CAA and ICH.^11^ Other monogenic variants identified by genome-wide association studies include *APOE*, *CR1*, *KCNK17*, *CETP*, *STYK1*, *COL4A2*, *NOTCH3*, *1q22*, and *17p12*.^12,13^ Sickle cell disease is associated with a high risk of both ischemic stroke and ICH, as repeated sickle crises modify cerebral vascaluture.^14^

The complex genetic predisposition to ICH may be best reflected by a polygenic risk score involving 2.6 million variants comprising 21 clinical attributes that determine either predisposing factors (eg, BP, renal function, and lipid profile) or underlying pathology (eg, cSVD).^15^

### Modifiable Predisposing Factors

Spontaneous ICH is not an inevitable consequence of aging. Many modifiable predisposing factors have been identified by rigorous large cohort and case-control studies examining multiple factors or systematic reviews of individual predisposing factors (Table S1). Most studies are restricted to high-income countries and sometimes group ICH with subarachnoid hemorrhage (collectively, hemorrhagic stroke) or subdural hemorrhage, which may weaken the strength of identified associations if predisposing factors are not shared by ICH with other intracranial hemorrhages.

Lifestyle exposures that predispose to ICH include high alcohol intake, extremes of weight, unhealthy diet, physical inactivity, and psychosocial stress (including stress at work and home, depression, and stressful life events, although the latter might also be a precipitating factor). The data on smoking are unclear: an association was present in a cohort of 1 million women^16^ but not in other case-control studies.^4,17^ The Global Burden of Disease study is not included in Table S1 because it measures disability-adjusted life years rather than incidence, uses a modeling approach, and reports population attributable fraction rather than risk ratios; it demonstrated a 15.4% (13.2%–17.7%) population attributable fraction for smoking for ICH, with high BP, air pollution, high sodium diet, and kidney dysfunction each contributing >10% of the population attributable fraction.^1^

The leading predisposing comorbidity is hypertension (including gestational hypertension), which has a consistent association in case-control and cohort studies (Table S1), a 56.4% (41.8%–67.7%) population attributable fraction in the Global Burden of Disease study, and a plausible biological mechanism due to the association between hypertension and cSVD.^18^ Chronic kidney disease is another prevalent predisposing factor that has been shown to be independent of hypertension in Mendelian randomization analysis.^19^ Chronic inflammation (eg, due to systemic lupus erythematosus and rheumatoid arthritis) or infection (eg, HIV) may lead to accelerated atherosclerosis, vasculitis, or vascular remodeling, all of which may increase ICH risk.^20^ Prior ischemic stroke is associated with ICH, perhaps due to shared underlying pathology^7^; although this article is generally concerned with first events, people with prior ICH have a high risk of recurrence.^21^ The mechanism for the association between migraine and ICH is unclear. Cardiac conditions such as atrial fibrillation may not directly cause pathology related to ICH; the association may be due to anticoagulant therapy precipitating bleeding in people with vascular disease. Lower LDL (low-density lipoprotein) cholesterol has a convincing epidemiological relationship with spontaneous ICH, and Mendelian randomization studies suggest a causal association, which might be mediated via endothelial fragility caused by smooth muscle cell necrosis.^22–24^

### Medicines

Several drugs are associated with ICH, but randomized controlled trials are usually too small to detect rare side effects (only 6 intracranial hemorrhages were reported in the 2900 patients in randomized trials comparing warfarin with placebo/open control for atrial fibrillation^25^). Nonetheless, studies have not found large or consistent associations between statin therapy or selective serotonin reuptake inhibitors and ICH.^26,27^ Of the nonsteroidal anti-inflammatory drugs, diclofenac and meloxicam may increase risk.^28^ Antiplatelet therapy shows a small, nonsignificant association with ICH (rate ratio, 1.32 [0.98–1.54]).^29^ Population-based cohorts suggest that the increased incidence of bleeding in people on anticoagulant drugs is highest in the first month after initiation.^30^ We hypothesize that oral anticoagulants are not predisposing factors but precipitating factors in people with underlying pathology and predisposing factors.

### Interaction of Predisposing Factors

Predisposing factors are likely to interact or be on common causal pathways. In the United States, Black and Hispanic people had a stronger association between hypertension and ICH than White people, but *APOE* allele ε2 or ε4 was only associated with ICH in White people, perhaps due to genetic or sociocultural factors.^7^ A prospective cohort study of 19 356 Japanese men found an interaction between alcohol and social support in risk of hemorrhagic stroke.^31^ Mediation analysis of a Mendelian randomization study found that 57% of the ICH risk associated with a genetic tendency to obesity was mediated by type 2 diabetes.^32^ However, we could not find any studies of associations or interactions between multiple long-term conditions and ICH incidence. A better understanding of interactions between predisposing factors could allow the identification of high-risk populations and improve preventative strategies.

## Pathologies Underlying Spontaneous ICH

Predisposing factors may mediate their effects by causing or modifying the severity of specific microvascular, macrovascular, hemodynamic, and neoplastic pathologies underlying ICH. Predisposing factors and pathology may interact.

### Microvascular Disorders

Most ICH is due to cSVD, which is extremely prevalent in older adults.^33^ Imaging features provide evidence of the presence of cSVD and its subtypes. Sporadic cSVD includes arteriolosclerosis, lipohyalinosis, and fibrinoid necrosis^18^ and, less often, CAA or rarer microangiopathies. The underlying cause of sporadic CAA is incompletely understood but involves β-amyloid deposition in cortical and leptomeningeal vessel walls, likely due to reduced peptide clearance,^34^ leading to lobar ICH in a small proportion of people.^33^ Known predisposing factors are age, *APOE* ε4 or ε2 genotype, and iatrogenic causes, including neurosurgical procedures and treatment with human growth hormone.^35,36^

Arteriolosclerosis affects small arteries and arterioles throughout the brain and is associated with hypertension. Specific genetic variants (eg, in the *NOTCH3* gene) are associated with cSVD and increased risk of ICH, particularly in people with other predisposing factors such as hypertension.^13^ Modifiable risk factors for cSVD, including obesity and hypertension, may predispose to chronic inflammation leading to remodeling of blood vessels.^37^ ICH location is a component of the Boston criteria for CAA, and epidemiological studies suggest different risk factor profiles for lobar ICH compared with nonlobar ICH.^17^ Deep ICH (where CAA does not occur) is usually due to arteriolosclerosis and is consequently often attributed to high BP. However, hypertension is associated with ICH in any location (although more prevalent with deep versus lobar ICH), and moderate-severe arteriolosclerosis underlies four-fifths of lobar ICH.^2,17^

### Disorders of Blood Vessel Function

Reversible cerebral vasoconstriction syndrome and posterior reversible encephalopathy syndrome have clinical and imaging overlap, suggesting shared pathophysiology. Up to a quarter of people with these syndromes develop ICH. Postmortem examination shows normal blood vessels.^38^ Possible mechanisms include failure of cerebral autoregulation, cerebrovascular tone, and endothelial function.^39^

### Macrovascular Disorders

Macrovascular abnormalities (aneurysms, arteriovenous malformations, dural arteriovenous fistulae, and cerebral cavernous malformations) are rare in the general population^40^ but together account for ≈10% of spontaneous ICH and can often be identified on angiographic imaging. No predisposing factors are known to affect the bleeding risk of arteriovenous malformations or cavernous malformations.^41,42^ Intracranial aneurysms occur sporadically; risk factors for rupture include hypertension, age, smoking, previous subarachnoid hemorrhage, aneurysm size and location, and geographic region.^43,44^ Mycotic aneurysms and septic arteritis occur secondary to infection, including bacterial endocarditis, dental infection, and cavernous sinus syndrome.^45^ Moyamoya disease is rare, although more common in East Asian populations, probably due to genetic differences.^46^ Moyamoya syndrome describes the imaging appearance of moyamoya disease secondary to another condition such as sickle cell disease. Cerebral vasculitis is an uncommon cause of ICH that may affect blood vessels of any size depending on the cause. Blood clots can lead to ICH: hemorrhage sometimes follows cerebral venous sinus thrombosis, as pressure builds in upstream vasculature; and arterial occlusion can disrupt vasculature, leading to hemorrhagic transformation of cerebral infarction.^47^

### Tumor and Infection

Brain tumors (either primary brain malignancy or metastases) can be highly angiogenic and present with ICH. Infections can cause bleeding either directly or indirectly through abscess formation, septic emboli from remote infection, or vasculitis secondary to, for example, herpes simplex encephalitis.

## Precipitating Factors for Spontaneous ICH

A precipitating factor may lead to spontaneous ICH by either causing hemorrhage from a vessel or modifying the severity of subclinical blood leakage. Not every ICH will have an identifiable precipitating factor, many of which are unavoidable, but some are modifiable and have clinical implications, particularly for people with underlying pathology.

A systematic review without meta-analysis concluded that in adults with hemophilia, severity of disease and hypertension were risk factors for ICH; it is plausible that, particularly in older adults, hypertension may lead to cSVD, and bleeding is precipitated by reduced coagulation function.^48^

There is no obvious mechanism for people on therapeutic doses of antithrombotic therapy to bleed spontaneously in the absence of underlying pathology. However, anticoagulant therapy might precipitate symptomatic ICH in a person with pathology and subclinical ICH (eg, cerebral microhemorrhage),^49,50^ supported by population-based studies that demonstrate that the risk of ICH is highest in the first 30 days after starting warfarin.^30^ This distinction between precipitating and predisposing factors is important, as ascertaining underlying pathology is important regardless of whether a person is on anticoagulant therapy. Similarly, ICH precipitated by thrombolytics administered for treatment of acute ischemic stroke can be remote from the area of infarction and is associated with underlying pathology of cerebral microbleeds and white matter hyperintensities on brain imaging.^51^

Fluctuations in BP and vascular tone may precipitate ICH. BP increases in the days and weeks before ICH.^52^ A population-based cohort study in England found a higher risk of ICH in the first 6 weeks postpartum among women of childbearing age (incidence rate ratio, 3.6 [95% CI, 1.5–8.7]) but not during antepartum or peripartum periods, perhaps related to preeclampsia and gestational hypertension.^53^

Activities that affect BP and vascular tone have been investigated using case-crossover designs, comparing activities over a period immediately before an event, to a period in the past. Anger, heavy exercise, sexual activity, valsalva maneuvers including defecation, cola and coffee consumption, flu-like disease, overeating, playing games, such as mahjong and chess, and death of a partner have all been identified as possible ICH precipitants using this study design.^54–59^ However, large odds ratios in some studies suggest recall bias influenced results; in one study, fewer than half of participants recalled having a flu-like illness in the past year, but people in Western Europe average 2.7 upper respiratory tract infections a year.^60^ A recent systematic review identified several statistically significant precipitating factors, including antiplatelet, anticoagulant, nonsteroidal anti-Inflammatory drug and antipsychotic use; anger; cola consumption; and defecation, although the risk estimates were imprecise and included studies that were heterogeneous both statistically and methodologically.^61^

Heavy alcohol intake increases the risk of ICH in the subsequent day and week.^62^ Stimulant drugs including cocaine and amphetamines can precipitate ICH and are important to consider in younger populations.^63,64^ Changes in environment, including increased air pollution and lower ambient temperature, are associated with small increases in incidence of ICH,^65,66^ and risk increases following several viral and bacterial infections.^67–70^

## Application of a Framework for the Cause of Spontaneous ICH

This extensive body of evidence about the many predisposing factors, underlying pathologies, and precipitating factors suggests that there is a complex interplay of these many factors in causing ICH (Figure). The factors listed in the Figure seem to be the most clinically and statistically significant factors according to current evidence (Supplemental Material), and they have implications for clinical practice and future research.

### Implications for Clinical Practice

The framework echoes the model of predisposing, precipitating, perpetuating, and protective factors for psychiatric disease and provides a clinically intuitive structure to classify cause, reflecting the complexity seen in clinical practice and epidemiological studies. It does not preclude interactions between predisposing factors, pathologies, and precipitating factors and does not require all categories to explain ICH in one person. Rather, the framework allows a holistic view of each patient, which recognizes that people often have multiple long-term conditions and other predisposing factors for ICH.

This framework encourages attribution of all of the potential causes that exist in one patient (eg, an octogenarian man, of lower socioeconomic status, exposed to high ambient air pollution, suffering psychosocial stress following a recent bereavement, with prior alcohol misuse, and a history of hypertension and atrial fibrillation for which he has been taking oral anticoagulation, who experiences lobar ICH with mixed CAA and deep perforating arteriopathy cSVD biomarkers on magnetic resonance imaging). The recognition and explanation of these multiple factors by physicians avoid a focus on one predisposing factor (eg, hypertension) that disregards others (eg, alcohol misuse), treats multiple underlying pathologies equally, and identifies all precipitating factors rather than just one (eg, anticoagulation). This explanation avoids value judgments about attribution and provides multiple modifiable approaches to the prevention of future major adverse cardiovascular events for patients.

### Implications for Research

Several classification systems for the cause of ICH have been proposed, aiming to stratify patients by survival, target treatment, or phenotype for clinical research (Table 1).^71–74^ However, none of these systems encompasses the spectrum of predisposing factors for ICH, nor reflects the complexity of their combination with underlying pathologies and predisposing factors on the causal pathway that is seen in most patients in clinical practice.

**Table 1.**
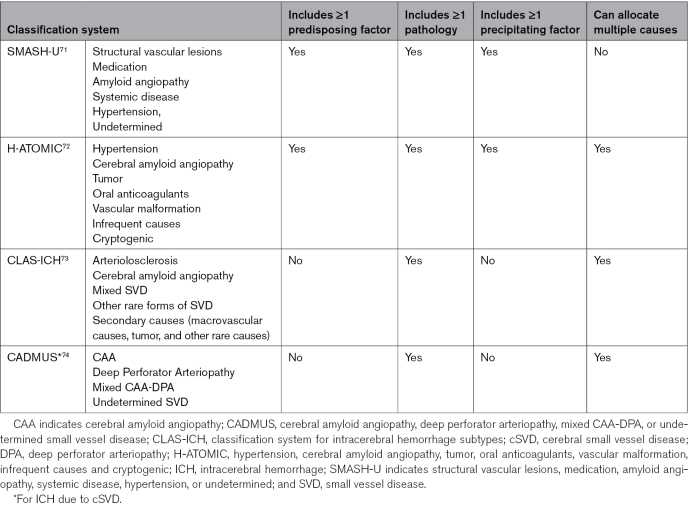
Published Causal Classification Systems for Intracerebral Hemorrhage

SMASH-U (structural vascular lesions, medication, amyloid angiopathy, systemic disease, hypertension, or undetermined) assigns a single category from a group of predisposing factors, precipitating factors, and pathological findings but does not allow allocation of multiple causes.^71^ In SMASH-U, hemorrhage location primarily determines underlying cSVD pathology (CAA or hypertension), a method now superseded by computed tomography and magnetic resonance imaging–based criteria for CAA. The categories were defined by the authors of this article and are associated with long-term survival. SMASH-U has since been modified with the addition of posterior reversible encephalopathy syndrome/reversible cerebral vasoconstriction syndrome for young adults.^75^ However, SMASH-U does not include several predisposing factors (such as alcohol, chronic kidney disease, or HIV), pathologies (such as cerebral vasculitis), and precipitating factors (such as infection and pregnancy).

H-ATOMIC (hypertension, cerebral amyloid angiopathy, tumour, oral anticoagulants, vascular malformation, infrequent causes and cryptogenic) is similar to SMASH-U but allows for attribution of multiple causes, with a degree of certainty (definite, probable, or possible) for each.^72^ The category definitions in H-ATOMIC are complex and, for hypertension, include raised BP in the 6 hours after ICH, a physiological response seen in three-quarters of patients.^76^ The system has been applied to patients in clinical practice: in a cohort of 439 people with ICH, most were attributed a cause, and only 2% were classified as cryptogenic. However, H-ATOMIC does not include several predisposing factors (such as chronic kidney disease and HIV) and precipitating factors (such as pregnancy) and classifies alcohol as an infrequent cause despite the high prevalence of its use and its association with ICH.

For both H-ATOMIC and SMASH-U, it is not clear how the list of causes or the criteria for ascertaining those causes was derived. H-ATOMIC and SMASH-U both include hypertension, a clear predisposing factor, but not other factors with consistent evidence and biological plausibility, such as alcohol use (perhaps due to the lack of primary studies when these classification systems were designed). H-ATOMIC and SMASH-U include oral anticoagulation, which is unlikely to be the sole cause. Despite their similarities, in a head-to-head comparison, SMASH-U and H-ATOMIC classified one-third of patients differently, usually because >1 potential cause was present.^77^

Two other classification systems, CLAS-ICH (classification system for ICH subtypes) and CADMUS (cerebral amyloid angiopathy, deep perforator arteriopathy [DPA], mixed CAA-DPA, or undetermined SVD), are restricted to classifying ICH attributed to cSVD on the basis of computed tomography and magnetic resonance imaging features, respectively, and can be used to identify CAA, non-CAA, and mixed cSVD on imaging.^73,74^ These systems build on the pathologically validated Boston criteria for CAA on magnetic resonance imaging and the Edinburgh criteria for CAA on computed tomography. CLAS-ICH and CADMUS have demonstrated reproducibility among different raters and cohorts and allow for multiple underlying pathologies, but neither incorporates predisposing factors to these pathologies or precipitating factors, providing only one dimension of the cause of a person’s ICH.

All 4 classification systems attempt to provide simple, practical approaches but do not reflect the number and variety of predisposing factors seen in patients in clinical practice, who usually have multiple long-term conditions (only H-ATOMIC applies to all ICH and allows multiple causes), and none reflects the complexity of their combination with underlying pathologies and precipitating factors on the causal pathway that is seen in most patients in clinical practice. Although reductionist approaches simplify statistical analysis, multivariable approaches and mediation analyses could dissect the multitude of factors contributing to the cause of ICH. The danger of simplifying attribution of cause is that these classification systems could lead to unintended cognitive biases in clinical practice, resulting in modifiable factors being overlooked.

### Future Directions and Conclusions

The framework suggests questions for future research into interactions between predisposing factors, precipitating factors, and pathology (Table 2). Answering these questions may require innovative research methods. ICH is a relatively common condition where research is hampered by difficulties in recruiting frail and unwell participants. Along with traditional approaches identifying causal pathways to ICH with animal models and randomized controlled trials, artificial intelligence techniques applied to large-scale, detailed data sets describing the many factors contributing to the cause of ICH could assist with prediction and stratification of those at risk. Combining artificial intelligence with causal inference techniques may produce the most clinically useful results for both prediction and treatment of ICH.^78^ Artificial intelligence analysis of brain imaging at scale can be linked to other population health data to enhance understanding of how risk factors are associated with underlying pathology and identify populations for more detailed cohort and interventional studies.^79^ Within these large data sets, clustering techniques such as latent class analysis could be used to align predisposing factors to underlying pathology (including markers of macrovascular and microvascular diseases, and anatomic location) and stratify based on prognosis.^80,81^ The conceptual approach and analytical techniques can incorporate new discoveries and guide further research into the mechanism of ICH and its prevention, prognosis, and treatment. Ultimately, research should aim to produce a model where people with ICH can be given personalized prevention and treatment depending on their underlying predisposing factors and pathology, which could be translated to clinical practice.

**Table 2.**
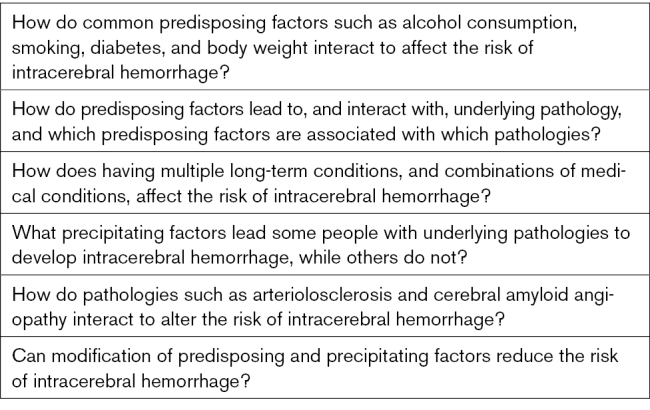
Questions for Future Research

Although our framework is inclusive and exposes knowledge gaps (specifically the interplay between predisposing factors, pathologies, and precipitating factors), it also has some limitations. First, our framework is not conveniently reductionist. Second, although there is extensive primary research on individual predisposing factors (Supplemental Material), residual confounding may affect these, and uncertainties remain around the interactions between predisposing factors that are common in the general population. Third, we do not know how the many genetic factors, lifestyle factors, and medical conditions interact to lead to underlying pathology and precipitate ICH or how having multiple long-term conditions or particular combinations of conditions affects risk.

Ultimately, future research could use this framework to derive and validate a more inclusive system for classifying the cause of ICH and test its association with prognosis or modification of interventions’ effects in clinical trials to add clinical utility by personalizing approaches to investigation, acute treatment, prevention of complications, and secondary prevention.

## Article Information

### Sources of Funding

A. Hosking is funded by a clinical research training fellowship from the UK Medical Research Council and The Stroke Association (grant MR/Z504051/1).

### Disclosures

A. Hosking reports grants from the UK Medical Research Council and the Stroke Association. Dr Samarasekera reports grants from the Stroke Association and NHS Research Scotland. Dr Moullaali reports grants from Scottish Heart and Arterial Risk Prevention (SHARP) and the British Heart Foundation. Dr Whiteley reports compensation from Bayer for consultant services; compensation from the University of Utrecht, The University of Manchester, the University of Oxford, and Calgary University for data and safety monitoring services; and grants from the Chief Scientist Office; compensation from UK Courts for expert witness services. Dr Murthy reports grants from the National Institutes of Health. Dr Gaist reports compensation from Bristol-Myers Squibb, Pfizer, and Bayer for other services. Dr Muñoz Venturelli reports grants from Agencia Nacional de Investigación y Desarrollo and Pfizer, and grants from Boehringer Ingelheim to others. Dr Cheng reports grants from the Science and Technology Commission of Shanghai Municipality, the Noncommunicable Chronic Diseases-National Science and Technology Major Project, the Shanghai Municipal Health Commission, and the National Natural Science Foundation of China. Dr Anderson reports compensation from Auzone Pharma, China, and AstraZeneca Australia for consultant services and grants from the National Health and Medical Research Council. Dr Shoamanesh reports compensation from Bayer for data and safety monitoring services; grants from Bayer, the Heart and Stroke Foundation of Canada, AstraZeneca, Daiichi Sankyo Company, Octapharma USA, Inc, the Canadian Institutes of Health Research, Bayer, Servier Affaires Medicales, Canadian Institutes of Health Research, and the National Institutes of Health; and compensation from AstraZeneca, Bayer, Takeda Pharmaceutical Company, and Daiichi Sankyo Company for consultant services.

Dr Al-Shahi Salman has had funding paid to his institution from the Chief Scientist Office (Scotland), the British Heart Foundation, Recursion Pharmaceuticals, NovoNordisk, European Stroke Organisation, and the UK Clinical Research Collaboration network of Registered Clinical Trials Units. The other authors report no conflicts.

### Supplemental Material

Search Strategies and Study Selection Criteria

Table S1

References 81–109

## References

[1] Collaborators GSRF. Global, regional, and national burden of stroke and its risk factors, 1990-2021: a systematic analysis for the Global Burden of Disease Study 2021. Lancet Neurol. 2024;23:973–1003. doi: 10.1016/S1474-4422(24)00369-7 39304265 10.1016/S1474-4422(24)00369-7 PMC12254192

[2] Rodrigues MA Samarasekera N Lerpiniere C Humphreys C McCarron MO White PM Nicoll JAR Sudlow CLM Cordonnier C Wardlaw JM. The Edinburgh CT and genetic diagnostic criteria for lobar intracerebral haemorrhage associated with cerebral amyloid angiopathy: model development and diagnostic test accuracy study. Lancet Neurol. 2018;17:232–240. doi: 10.1016/S1474-4422(18)30006-1 29331631 10.1016/S1474-4422(18)30006-1 PMC5818029

[3] O’Donnell MJ Chin SL Rangarajan S Xavier D Liu L Zhang H Rao-Melacini P Zhang X Pais P Agapay S; INTERSTROKE investigators. Global and regional effects of potentially modifiable risk factors associated with acute stroke in 32 countries (INTERSTROKE): a case-control study. Lancet. 2016;388:761–775. doi: 10.1016/S0140-6736(16)30506-2 27431356 10.1016/S0140-6736(16)30506-2

[4] O’Donnell MJ Chin SL Rangarajan S Xavier D Liu L Zhang H Rao-Melacini P Zhang X Pais P Agapay S; INTERSTROKE investigators. Global and regional effects of potentially modifiable risk factors associated with acute stroke in 32 countries (INTERSTROKE): a case-control study. Lancet. 2016;388:761–775. doi: 10.1016/S0140-6736(16)30506-2 27431356 10.1016/S0140-6736(16)30506-2

[5] Bray BD Paley L Hoffman A James M Gompertz P Wolfe CDA Hemingway H Rudd AG, Collaboration S. Socioeconomic disparities in first stroke incidence, quality of care, and survival: a nationwide registry-based cohort study of 44 million adults in England. Lancet Public Health. 2018;3:e185–e193. doi: 10.1016/S2468-2667(18)30030-6 29550372 10.1016/S2468-2667(18)30030-6 PMC5887080

[6] van Asch CJJ Luitse MJA Rinkel GJE van der Tweel I Algra A Klijn CJM. Incidence, case fatality, and functional outcome of intracerebral haemorrhage over time, according to age, sex, and ethnic origin: a systematic review and meta-analysis. Lancet Neurol. 2010;9:167–176. doi: 10.1016/S1474-4422(09)70340-0 20056489 10.1016/S1474-4422(09)70340-0

[7] Kittner SJ Sekar P Comeau ME Anderson CD Parikh GY Tavarez T Flaherty ML Testai FD Frankel MR James ML. Ethnic and racial variation in intracerebral hemorrhage risk factors and risk factor burden. JAMA Netw Open. 2021;4:e2121921–e2121921. doi: 10.1001/jamanetworkopen.2021.21921 34424302 10.1001/jamanetworkopen.2021.21921 PMC8383133

[8] Poorthuis MHF Algra AM Algra A Kappelle LJ Klijn CJM. Female- and male-specific risk factors for stroke. JAMA Neurol. 2017;74:75–81. doi: 10.1001/jamaneurol.2016.3482 27842176 10.1001/jamaneurol.2016.3482

[9] Biffi A. Main features of hereditary cerebral amyloid angiopathies: a systematic review. Cereb Circ Cogn Behav. 2022;3:100124–100124. doi: 10.1016/j.cccb.2022.100124 36324420 10.1016/j.cccb.2022.100124 PMC9616336

[10] Flemming KD Smith E Marchuk D Derry WB. Familial cerebral cavernous malformations. In: Adam MP Bick S Mirzaa GM, , eds. GeneReviews®. Seattle (WA): University of Washington. 2023. Accessed January 23, 2024. https://www.ncbi.nlm.nih.gov/books/NBK1293/20301470

[11] Sobey CG Judkins CP Sundararajan V Phan TG Drummond GR Srikanth VK. Risk of major cardiovascular events in people with Down syndrome. PLoS One. 2015;10:e0137093–e0137093. doi: 10.1371/journal.pone.0137093 26421620 10.1371/journal.pone.0137093 PMC4589343

[12] Wahab KW Tiwari HK Ovbiagele B Sarfo F Akinyemi R Traylor M Rotimi C Markus HS Owolabi M. Genetic risk of spontaneous intracerebral hemorrhage: systematic review and future directions. J Neurol Sci. 2019;407:116526–116526. doi: 10.1016/j.jns.2019.116526 31669726 10.1016/j.jns.2019.116526 PMC7413646

[13] Cho BPH Harshfield EL Al-Thani M Tozer DJ Bell S Markus HS. Association of vascular risk factors and genetic factors with penetrance of variants causing monogenic stroke. JAMA Neurol. 2022;79:1303–1311. doi: 10.1001/jamaneurol.2022.3832 36300346 10.1001/jamaneurol.2022.3832 PMC9614680

[14] Strouse JJ Lanzkron S Urrutia V. The epidemiology, evaluation and treatment of stroke in adults with sickle cell disease. Expert Rev Hematol. 2011;4:597–606. doi: 10.1586/ehm.11.61 22077524 10.1586/ehm.11.61 PMC3267235

[15] Myserlis EP Georgakis MK Demel SL Sekar P Chung J Malik R Hyacinth HI Comeau ME Falcone GJ Langefeld CD. A genomic risk score identifies individuals at high risk for intracerebral hemorrhage. Stroke. 2023;54:973–982. doi: 10.1161/STROKEAHA.122.041701 36799223 10.1161/STROKEAHA.122.041701 PMC10050100

[16] Price AJ Wright FL Green J Balkwill A Kan SW Yang TO Floud S Kroll ME Simpson R Sudlow CLM. Differences in risk factors for 3 types of stroke: UK prospective study and meta-analyses. Neurology. 2018;90:e298–e306. doi: 10.1212/WNL.0000000000004856 29321237 10.1212/WNL.0000000000004856 PMC5798656

[17] Jolink WMT Wiegertjes K Rinkel GJE Algra A de Leeuw F-E Klijn CJM. Location-specific risk factors for intracerebral hemorrhage. Neurology. 2020;95:e1807–e1818. doi: 10.1212/WNL.0000000000010418 32690784 10.1212/WNL.0000000000010418

[18] Wardlaw JM Smith C Dichgans M. Small vessel disease: mechanisms and clinical implications. Lancet Neurol. 2019;18:684–696. doi: 10.1016/S1474-4422(19)30079-1 31097385 10.1016/S1474-4422(19)30079-1

[19] Vanent KN Leasure AC Acosta JN Kuohn LR Woo D Murthy SB Kamel H Messé SR Mullen MT Cohen JB. Association of chronic kidney disease with risk of intracerebral hemorrhage. JAMA Neurol. 2022;79:911–918. doi: 10.1001/jamaneurol.2022.2299 35969388 10.1001/jamaneurol.2022.2299 PMC9379821

[20] Benjamin L Khoo S. HIV infection and stroke. In: Handbook of Clinical Neurology. Elsevier; 2018:187–200. 10.1016/B978-0-444-63849-6.00015-3 29604976

[21] Li L Poon MTC Samarasekera NE Perry LA Moullaali TJ Rodrigues MA Loan JJM Stephen J Lerpiniere C Tuna MA. Risks of recurrent stroke and all serious vascular events after spontaneous intracerebral haemorrhage: pooled analyses of two population-based studies. Lancet Neurol. 2021;20:437–447. doi: 10.1016/S1474-4422(21)00075-2 34022170 10.1016/S1474-4422(21)00075-2 PMC8134058

[22] Falcone GJ Kirsch E Acosta JN Noche RB Leasure A Marini S Chung J Selim M Meschia JF Brown DL; International Stroke Genetics Consortium. Genetically elevated LDL associates with lower risk of intracerebral hemorrhage. Ann Neurol. 2020;88:56–66. doi: 10.1002/ana.25740 32277781 10.1002/ana.25740 PMC7523882

[23] Sun L Clarke R Bennett D Guo Y Walters RG Hill M Parish S Millwood IY Bian Z Chen Y; China Kadoorie Biobank Collaborative Group. Causal associations of blood lipids with risk of ischemic stroke and intracerebral hemorrhage in Chinese adults. Nat Med. 2019;25:569–574. doi: 10.1038/s41591-019-0366-x 30858617 10.1038/s41591-019-0366-x PMC6795549

[24] Ma C Na M Neumann S Gao X. Low-Density lipoprotein cholesterol and risk of hemorrhagic stroke: a systematic review and dose-response meta-analysis of prospective studies. Curr Atheroscler Rep. 2019;21:52. doi: 10.1007/s11883-019-0815-5 31748963 10.1007/s11883-019-0815-5

[25] Hart RG Benavente O McBride R Pearce LA. Antithrombotic therapy to prevent stroke in patients with atrial fibrillation. Ann Intern Med. 1999;131:492–501. doi: 10.7326/0003-4819-131-7-199910050-00003 10507957 10.7326/0003-4819-131-7-199910050-00003

[26] Jensen MP Ziff OJ Banerjee G Ambler G Werring DJ. The impact of selective serotonin reuptake inhibitors on the risk of intracranial haemorrhage: a systematic review and meta-analysis. Eur Stroke J. 2019;4:144–152. doi: 10.1177/2396987319827211 31259262 10.1177/2396987319827211 PMC6591760

[27] Cholesterol Treatment Trialists’ C Baigent C Blackwell L Emberson J Holland LE Reith C Bhala N Peto R Barnes EH Keech A. Efficacy and safety of more intensive lowering of LDL cholesterol: a meta-analysis of data from 170,000 participants in 26 randomised trials. Lancet. 2010;376:1670–1681. doi: 10.1016/s0140-6736(10)61350-5 21067804 10.1016/S0140-6736(10)61350-5 PMC2988224

[28] Ungprasert P Matteson EL Thongprayoon C. Nonaspirin nonsteroidal anti-inflammatory drugs and risk of hemorrhagic stroke. Stroke. 2016;47:356–364. doi: 10.1161/STROKEAHA.115.011678 26670086 10.1161/STROKEAHA.115.011678

[29] Huang W-Y Saver JL Wu Y-L Lin C-J Lee M Ovbiagele B. Frequency of intracranial hemorrhage with low-dose aspirin in individuals without symptomatic cardiovascular disease: a systematic review and meta-analysis. JAMA Neurol. 2019;76:906–914. doi: 10.1001/jamaneurol.2019.1120 31081871 10.1001/jamaneurol.2019.1120 PMC6515567

[30] Gonzalez-Perez A Gaist D Garcia Rodriguez LA. Warfarin and absolute risk of hemorrhagic stroke. CMAJ. 2013;185:687–687. doi: 10.1503/cmaj.113-2115 10.1503/cmaj.113-2115 PMC3652941 23670867

[31] Ikehara S Iso H Yamagishi K Yamamoto S Inoue M Tsugane S; JPHC Study Group. Alcohol consumption, social support, and risk of stroke and coronary heart disease among Japanese men: the JPHC study. Alcohol Clin Exp Res. 2009;33:1025–1032. doi: 10.1111/j.1530-0277.2009.00923.x 19302085 10.1111/j.1530-0277.2009.00923.x

[32] Larsson SC Chen J Gill D Burgess S Yuan S. Risk factors for intracerebral hemorrhage: genome-wide association study and mendelian randomization analyses. Stroke. 2024;55:1582–1591. doi: 10.1161/STROKEAHA.124.046249 38716647 10.1161/STROKEAHA.124.046249 PMC11122740

[33] Samarasekera N Smith C Al-Shahi Salman R. The association between cerebral amyloid angiopathy and intracerebral haemorrhage: systematic review and meta-analysis. J Neurol Neurosurg Psychiatry. 2011;83:275–281. doi: 10.1136/jnnp-2011-300371 22056966 10.1136/jnnp-2011-300371

[34] Greenberg SM Bacskai BJ Hernandez-Guillamon M Pruzin J Sperling R van Veluw SJ. Cerebral amyloid angiopathy and Alzheimer disease - one peptide, two pathways. Nat Rev Neurol. 2020;16:30–42. doi: 10.1038/s41582-019-0281-2 31827267 10.1038/s41582-019-0281-2 PMC7268202

[35] Charidimou A Boulouis G Gurol ME Ayata C Bacskai BJ Frosch MP Viswanathan A Greenberg SM. Emerging concepts in sporadic cerebral amyloid angiopathy. Brain. 2017;140:1829–1850. doi: 10.1093/brain/awx047 28334869 10.1093/brain/awx047 PMC6059159

[36] Banerjee G Samra K Adams ME Jaunmuktane Z Parry-Jones AR Grieve J Toma AK Farmer SF Sylvester R Houlden H. Iatrogenic cerebral amyloid angiopathy: an emerging clinical phenomenon. J Neurol Neurosurg Psychiatry. 2022;93:693–700. doi: 10.1136/jnnp-2022-328792 10.1136/jnnp-2022-328792 35577510

[37] Evans LE Taylor JL Smith CJ Pritchard HAT Greenstein AS Allan SM. Cardiovascular comorbidities, inflammation, and cerebral small vessel disease. Cardiovasc Res. 2021;117:2575–2588. doi: 10.1093/cvr/cvab284 34499123 10.1093/cvr/cvab284

[38] Singhal AB. Posterior reversible encephalopathy syndrome and reversible cerebral vasoconstriction syndrome as syndromes of cerebrovascular dysregulation. Continuum (Minneapolis, Minn). 2021;27:1301–1320. doi: 10.1212/CON.0000000000001037 34618761 10.1212/CON.0000000000001037

[39] Fugate JE Rabinstein AA. Posterior reversible encephalopathy syndrome: clinical and radiological manifestations, pathophysiology, and outstanding questions. Lancet Neurol. 2015;14:914–925. doi: 10.1016/S1474-4422(15)00111-8 26184985 10.1016/S1474-4422(15)00111-8

[40] Morris Z Whiteley WN Longstreth WT Jr Weber F Lee Y-C Tsushima Y Alphs H Ladd SC Warlow C Wardlaw JM. Incidental findings on brain magnetic resonance imaging: systematic review and meta-analysis. BMJ. 2009;339:b3016–b3016. doi: 10.1136/bmj.b3016 19687093 10.1136/bmj.b3016 PMC2728201

[41] Kim H Al-Shahi Salman R McCulloch CE Stapf C Young WL Coinvestigators M. Untreated brain arteriovenous malformation: patient-level meta-analysis of hemorrhage predictors. Neurology. 2014;83:590–597. doi: 10.1212/WNL.0000000000000688 25015366 10.1212/WNL.0000000000000688 PMC4141996

[42] Horne M Flemming K Su IC Stapf C Brown R Christianson T Agid R terBrugge K Willinsky R Maxwell S. Clinical course of untreated cerebral cavernous malformations: individual patient data meta-analysis. Lancet Neurol. 2016;15:166–173. doi: 10.1016/S1474-4422(15)00303-8 26654287 10.1016/S1474-4422(15)00303-8 PMC4710581

[43] Backes D Rinkel GJE Laban KG Algra A Vergouwen MDI. Patient- and aneurysm-specific risk factors for intracranial aneurysm growth. Stroke. 2016;47:951–957. doi: 10.1161/strokeaha.115.012162 26906920 10.1161/STROKEAHA.115.012162

[44] Greving JP Wermer MJH Brown RD Morita A Juvela S Yonekura M Ishibashi T Torner JC Nakayama T Rinkel GJE. Development of the PHASES score for prediction of risk of rupture of intracranial aneurysms: a pooled analysis of six prospective cohort studies. Lancet Neurol. 2014;13:59–66. doi: 10.1016/S1474-4422(13)70263-1 24290159 10.1016/S1474-4422(13)70263-1

[45] Ducruet AF Hickman ZL Zacharia BE Narula R Grobelny BT Gorski J Connolly ES. Intracranial infectious aneurysms: a comprehensive review. Neurosurg Rev. 2009;33:37–46. doi: 10.1007/s10143-009-0233-1 19838745 10.1007/s10143-009-0233-1

[46] Mertens R Graupera M Gerhardt H Bersano A Tournier-Lasserve E Mensah MA Mundlos S Vajkoczy P. The genetic basis of moyamoya disease. Transl Stroke Res. 2022;13:25–45. doi: 10.1007/s12975-021-00940-2 34529262 10.1007/s12975-021-00940-2 PMC8766392

[47] Ahlqvist J. Stress-related intracerebral hemorrhage and the Water-Hammer effect. Stroke. 2001;32:275–278. doi: 10.1161/01.str.32.1.275-a 10.1161/01.str.32.1.275-a 11136950

[48] Zanon E Pasca S. Intracranial haemorrhage in children and adults with haemophilia A and B: a literature review of the last 20 years. Blood Transfus. 2019;17:378–384. doi: 10.2450/2019.0253-18 30747705 10.2450/2019.0253-18 PMC6774931

[49] Wilson D Ambler G Shakeshaft C Brown MM Charidimou A Al-Shahi Salman R Lip GYH Cohen H Banerjee G Houlden H; CROMIS-2 Collaborators. Cerebral microbleeds and intracranial haemorrhage risk in patients anticoagulated for atrial fibrillation after acute ischaemic stroke or transient ischaemic attack (CROMIS-2): a multicentre observational cohort study. Lancet Neurol. 2018;17:539–547. doi: 10.1016/S1474-4422(18)30145-5 29778365 10.1016/S1474-4422(18)30145-5 PMC5956310

[50] Seiffge DJ Goeldlin MB Tatlisumak T Lyrer P Fischer U Engelter ST Werring DJ. Meta-analysis of haematoma volume, haematoma expansion and mortality in intracerebral haemorrhage associated with oral anticoagulant use. J Neurol. 2019;266:3126–3135. doi: 10.1007/s00415-019-09536-1 31541341 10.1007/s00415-019-09536-1 PMC6851029

[51] Prats-Sanchez L Martínez-Domeño A Camps-Renom P Delgado-Mederos R Guisado-Alonso D Marín R Dorado L Rudilosso S Gómez-González A Purroy F. Risk factors are different for deep and lobar remote hemorrhages after intravenous thrombolysis. PLoS One. 2017;12:e0178284–e0178284. doi: 10.1371/journal.pone.0178284 28640874 10.1371/journal.pone.0178284 PMC5480833

[52] Fischer U Cooney MT Bull LM Silver LE Chalmers J Anderson CS Mehta Z Rothwell PM. Acute post-stroke blood pressure relative to premorbid levels in intracerebral haemorrhage versus major ischaemic stroke: a population-based study. Lancet Neurol. 2014;13:374–384. doi: 10.1016/S1474-4422(14)70031-6 24582530 10.1016/S1474-4422(14)70031-6 PMC4238109

[53] Ban L Sprigg N Abdul Sultan A Nelson-Piercy C Bath PM Ludvigsson JF Stephansson O Tata LJ. Incidence of first stroke in pregnant and nonpregnant women of childbearing age: a population-based cohort study from England. J Am Heart Assoc. 2017;6:e004601. doi: 10.1161/JAHA.116.004601 28432074 10.1161/JAHA.116.004601 PMC5532991

[54] Smyth A O’Donnell M Hankey GJ Rangarajan S Lopez-Jaramillo P Xavier D Zhang H Canavan M Damasceno A Langhorne P; INTERSTROKE Investigators. Anger or emotional upset and heavy physical exertion as triggers of stroke: the INTERSTROKE study. Eur Heart J. 2022;43:202–209. doi: 10.1093/eurheartj/ehab738 34850877 10.1093/eurheartj/ehab738 PMC10503880

[55] van Etten ES Kaushik K Jolink WMT Koemans EA Ekker MS Rasing I Voigt S Schreuder FHBM Cannegieter SC Rinkel GJE. Trigger factors for spontaneous intracerebral hemorrhage: a case-crossover study. Stroke. 2022;53:1692–1699. doi: 10.1161/STROKEAHA.121.036233 34911344 10.1161/STROKEAHA.121.036233

[56] Sallinen H Putaala J Strbian D. Triggering factors in non-traumatic intracerebral hemorrhage. J Stroke Cerebrovasc Dis. 2020;29:104921. doi: 10.1016/j.jstrokecerebrovasdis.2020.104921 32689642 10.1016/j.jstrokecerebrovasdis.2020.104921

[57] Ekker MS Verhoeven JI Rensink KML Schellekens MMI Boot EM van Alebeek ME Brouwers PJAM Arntz RM van Dijk GW Gons RAR. Trigger factors for stroke in young adults. Neurology. 2023;100:e49–e61. doi: 10.1212/WNL.0000000000201341 36127143 10.1212/WNL.0000000000201341

[58] Fenger-Grøn M Paulsen Møller I Schou Pedersen H Frost L Sandbæk A Davydow DS Johnsen SP Vinter N. Death of a partner and risks of ischemic stroke and intracerebral hemorrhage: a nationwide Danish matched cohort study. J Am Heart Assoc. 2020;9:e018763–e018763. doi: 10.1161/JAHA.120.018763 33198551 10.1161/JAHA.120.018763 PMC7763796

[59] Liu J Luo C Hu C Guo Y Cao F Li Y Yuan D Jiang W Yan J. Behavioral trigger factors for hemorrhagic stroke: a case-crossover study. Postgrad Med J. 2023;99:1013–1019. doi: 10.1093/postmj/qgad038 37209147 10.1093/postmj/qgad038

[60] Jin X Ren J Li R Gao Y Zhang H Li J Zhang J Wang X Wang G. Global burden of upper respiratory infections in 204 countries and territories, from 1990 to 2019. EClinicalMedicine. 2021;37:100986–100986. doi: 10.1016/j.eclinm.2021.100986 34386754 10.1016/j.eclinm.2021.100986 PMC8343248

[61] Liu J Luo C Guo Y Cao F Yan J. Individual trigger factors for hemorrhagic stroke: Evidence from case-crossover and self-controlled case series studies. Eur Stroke J. 2023;8:808–818. doi: 10.1177/23969873231173285 37641550 10.1177/23969873231173285 PMC10472950

[62] Mostofsky E Chahal HS Mukamal KJ Rimm EB Mittleman MA. Alcohol and immediate risk of cardiovascular events: a systematic review and dose-response meta-analysis. Circulation. 2016;133:979–987. doi: 10.1161/CIRCULATIONAHA.115.019743 26936862 10.1161/CIRCULATIONAHA.115.019743 PMC4783255

[63] Rendon LF Malta S Leung J Badenes R Nozari A Bilotta F. Cocaine and ischemic or hemorrhagic stroke: a systematic review and meta-analysis of clinical evidence. J Clin Med. 2023;12:5207. doi: 10.3390/jcm12165207 37629248 10.3390/jcm12165207 PMC10455873

[64] Indave BI Sordo L Bravo MJ Sarasa-Renedo A Fernández‐Balbuena S De la Fuente L Sonego M Barrio G. Risk of stroke in prescription and other amphetamine‐type stimulants use: a systematic review. Drug Alcohol Rev. 2017;37:56–69. doi: 10.1111/dar.12559 28485090 10.1111/dar.12559

[65] Guo Y Luo C Cao F Liu J Yan J. Short-term environmental triggers of hemorrhagic stroke. Ecotoxicol Environ Saf. 2023;265:115508. doi: 10.1016/j.ecoenv.2023.115508 37774546 10.1016/j.ecoenv.2023.115508

[66] Rowland ST Chillrud LG Boehme AK Wilson A Rush J Just AC Kioumourtzoglou M-A. Can weather help explain ‘why now?’: the potential role of hourly temperature as a stroke trigger. Environ Res. 2022;207:112229–112229. doi: 10.1016/j.envres.2021.112229 34699760 10.1016/j.envres.2021.112229 PMC8810591

[67] Forbes HJ Williamson E Benjamin L Breuer J Brown MM Langan SM Minassian C Smeeth L Thomas SL Warren-Gash C. Association of herpesviruses and stroke: systematic review and meta-analysis. PLoS One. 2018;13:e0206163–e0206163. doi: 10.1371/journal.pone.0206163 30462656 10.1371/journal.pone.0206163 PMC6248930

[68] Boehme AK Ranawat P Luna J Kamel H Elkind MSV. Risk of acute stroke after hospitalization for sepsis: a case-crossover study. Stroke. 2017;48:574–580. doi: 10.1161/STROKEAHA.116.016162 28196938 10.1161/STROKEAHA.116.016162 PMC5338564

[69] Sebastian S Stein LK Dhamoon MS. Infection as a stroke trigger. Stroke. 2019;50:2216–2218. doi: 10.1161/STROKEAHA.119.025872 31242826 10.1161/STROKEAHA.119.025872

[70] Wei K-C Sy C-L Wang W-H Wu C-L Chang S-H Huang Y-T. Major acute cardiovascular events after dengue infection-A population-based observational study. PLoS NeglTrop Dis. 2022;16:e0010134–e0010134. doi: 10.1371/journal.pntd.0010134 10.1371/journal.pntd.0010134 PMC8853534 35130277

[71] Meretoja A Strbian D Putaala J Curtze S Haapaniemi E Mustanoja S Sairanen T Satopää J Silvennoinen H Niemelä M. SMASH-U. Stroke. 2012;43:2592–2597. doi: 10.1161/STROKEAHA.112.661603 22858729 10.1161/STROKEAHA.112.661603

[72] Martí-Fàbregas J Prats-Sánchez L Martínez-Domeño A Camps-Renom P Marín R Jiménez-Xarrié E Fuentes B Dorado L Purroy F Arias-Rivas S. The H-ATOMIC criteria for the etiologic classification of patients with intracerebral hemorrhage. PLoS One. 2016;11:e0156992–e0156992. doi: 10.1371/journal.pone.0156992 27275863 10.1371/journal.pone.0156992 PMC4898692

[73] Raposo N Zanon Zotin MC Seiffge DJ Li Q Goeldlin MB Charidimou A Shoamanesh A Jäger HR Cordonnier C Klijn CJ. A causal classification system for intracerebral hemorrhage subtypes. Ann Neurol. 2023;93:16–28. doi: 10.1002/ana.26519 36197294 10.1002/ana.26519 PMC9839566

[74] Goeldlin MB Mueller M Siepen BM Zhang W Ozkan H Locatelli M Du Y Valenzuela W Radojewski P Hakim A; for Swiss Stroke Registry Investigators and SIGNAL Investigators. CADMUS: a novel MRI-based classification of spontaneous intracerebral hemorrhage associated with cerebral small vessel disease. Neurology. 2024;102:e207977–e207977. doi: 10.1212/WNL.0000000000207977 38165372 10.1212/WNL.0000000000207977 PMC10834115

[75] Sariyeva M Haghighi N Mitchell A Booker WA Petersen NH Shields AD Ghoshal S Agarwal S Park S Claassen J. Primary and secondary intracerebral hemorrhage in pregnant and nonpregnant young adults by SMASH-UP criteria. J Am Heart Assoc. 2024;13:e034032–e034032. doi: 10.1161/JAHA.123.034032 38533990 10.1161/JAHA.123.034032 PMC11179753

[76] Qureshi AI Ezzeddine MA Nasar A Suri MFK Kirmani JF Hussein HM Divani AA Reddi AS. Prevalence of elevated blood pressure in 563,704 adult patients with stroke presenting to the ED in the United States. Am J Emerg Med. 2007;25:32–38. doi: 10.1016/j.ajem.2006.07.008 17157679 10.1016/j.ajem.2006.07.008 PMC2443694

[77] Martí-Fàbregas J Prats-Sánchez L Guisado-Alonso D Martínez-Domeño A Delgado-Mederos R Camps-Renom P. SMASH-U versus H-ATOMIC: a head-to-head comparison for the etiologic classification of intracerebral hemorrhage. J Stroke Cerebrovasc Dis. 2018;27:2375–2380. doi: 10.1016/j.jstrokecerebrovasdis.2018.04.026 29779884 10.1016/j.jstrokecerebrovasdis.2018.04.026

[78] Hunter DJ Holmes C. Where medical statistics meets artificial intelligence. N Engl J Med. 2023;389:1211–1219. doi: 10.1056/NEJMra2212850 37754286 10.1056/NEJMra2212850

[79] JDCteam. 1.6 million brain scans to be analysed under SCAN-DAN Project. 2024. Accessed April 8, 2025. https://journalofdementiacare.co.uk/brain-scans-analysed-scan-dan

[80] Calvin CM Conroy MC Moore SF Kuzma E Littlejohns TJ. Association of multimorbidity, disease clusters, and modification by genetic factors with risk of dementia. JAMA Netw Open. 2022;5:e2232124–e2232124. doi: 10.1001/jamanetworkopen.2022.32124 36125811 10.1001/jamanetworkopen.2022.32124 PMC9490497

[81] Chapfuwa P Li C Mehta N Carin L Henao R. Survival cluster analysis. Proceedings of the ACM Conference on Health, Inference, and Learning. ACM. 2020;60–68. doi: 10.1145/3368555.3384465

[82] Zhao K Li J Du C Zhang Q Guo Y Yang M. Ambient fine particulate matter of diameter ≤ 2.5 μm and risk of hemorrhagic stroke: a systemic review and meta-analysis of cohort studies. Environ Sci Pollut Res Int. 2021;28:20970–20980. doi: 10.1007/s11356-021-13074-7 33694113 10.1007/s11356-021-13074-7 PMC8106587

[83] Fu W Liu Y Yan S Wen J Zhang J Zhang P Zou L. The association of noise exposure with stroke incidence and mortality: a systematic review and dose-response meta-analysis of cohort studies. Environ Res. 2022;215:114249. doi: 10.1016/j.envres.2022.114249 36058275 10.1016/j.envres.2022.114249

[84] Larsson SC Wallin A Wolk A Markus HS. Differing association of alcohol consumption with different stroke types: a systematic review and meta-analysis. BMC Med. 2016;14:178–178. doi: 10.1186/s12916-016-0721-4 27881167 10.1186/s12916-016-0721-4 PMC5121939

[85] Hu D Huang J Wang Y Zhang D Qu Y. Fruits and vegetables consumption and risk of stroke. Stroke. 2014;45:1613–1619. doi: 10.1161/STROKEAHA.114.004836 24811336 10.1161/STROKEAHA.114.004836

[86] Lee CD Folsom AR Blair SN. Physical activity and stroke risk. Stroke. 2003;34:2475–2481. doi: 10.1161/01.STR.0000091843.02517.9D 14500932 10.1161/01.STR.0000091843.02517.9D

[87] McCarthy CE Yusuf S Judge C Alvarez-Iglesias A Hankey GJ Oveisgharan S Damasceno A Iversen HK Rosengren A Avezum A; for INTERSTROKE. Sleep patterns and the risk of acute stroke: results from the INTERSTROKE international case-control study. Neurology. 2023;100:e2191–e2203. doi: 10.1212/WNL.0000000000207249 37019662 10.1212/WNL.0000000000207249 PMC10238154

[88] Pillay P Lewington S Taylor H Lacey B Carter J. Adiposity, body fat distribution, and risk of major stroke types among adults in the United Kingdom. JAMA Netw Open. 2022;5:e2246613–e2246613. doi: 10.1001/jamanetworkopen.2022.46613 36515951 10.1001/jamanetworkopen.2022.46613 PMC9856404

[89] Shiozawa M Kaneko H Itoh H Morita K Okada A Matsuoka S Kiriyama H Kamon T Fujiu K Michihata N. Association of body mass index with ischemic and hemorrhagic stroke. Nutrients. 2021;13:2343. doi: 10.3390/nu13072343 34371853 10.3390/nu13072343 PMC8308685

[90] Chen Z Iona A Parish S Chen Y Guo Y Bragg F Yang L Bian Z Holmes MV Lewington S; China Kadoorie Biobank Collaborative Group. Adiposity and risk of ischaemic and haemorrhagic stroke in 0·5 million Chinese men and women: a prospective cohort study. Lancet Glob Health. 2018;6:e630–e640. doi: 10.1016/S2214-109X(18)30216-X 29773119 10.1016/S2214-109X(18)30216-X PMC5960068

[91] Kroll ME Green J Beral V Sudlow CLM Brown A Kirichek O Price A Yang TO Reeves GK Million Women Study C. Adiposity and ischemic and hemorrhagic stroke: prospective study in women and meta-analysis. Neurology. 2016;87:1473–1481. doi: 10.1212/wnl.0000000000003171 27605176 10.1212/WNL.0000000000003171 PMC5075975

[92] Zou X Wang L Xiao L Xu Z Yao T Shen M Zeng Y Zhang L. Deciphering the irregular risk of stroke increased by obesity classes: a stratified Mendelian randomization study. Front Endocrinol (Lausanne). 2021;12:750999–750999. doi: 10.3389/fendo.2021.750999 34925231 10.3389/fendo.2021.750999 PMC8671740

[93] Waziry R Chibnik LB Bos D Ikram MK Hofman A. Risk of hemorrhagic and ischemic stroke in patients with Alzheimer disease: a synthesis of the literature. Neurology. 2020;94:265–272. doi: 10.1212/WNL.0000000000008924 31949087 10.1212/WNL.0000000000008924 PMC7136067

[94] Liu W Ma W Liu H Li C Zhang Y Liu J Liang Y Zhang S Wu Z Zang C. Stroke risk in arthritis: a systematic review and meta-analysis of cohort studies. PLoS One. 2021;16:e0248564–e0248564. doi: 10.1371/journal.pone.0248564 33725018 10.1371/journal.pone.0248564 PMC7963101

[95] Odutayo A Wong CX Hsiao AJ Hopewell S Altman DG Emdin CA. Atrial fibrillation and risks of cardiovascular disease, renal disease, and death: systematic review and meta-analysis. BMJ. 2016;354:i4482. doi: 10.1136/bmj.i4482 27599725 10.1136/bmj.i4482

[96] Turner M Murchie P Derby S Ong AY Walji L McLernon D Macleod M-J Adam R. Is stroke incidence increased in survivors of adult cancers? A systematic review and meta-analysis. J Cancer Surviv. 2022;16:1414–1448. doi: 10.1007/s11764-021-01122-7 34739710 10.1007/s11764-021-01122-7 PMC9630245

[97] Zheng K Yoshida EM Tacke F Li Y Guo X Qi X. Risk of stroke in liver cirrhosis. J Clin Gastroenterol. 2020;54:96–105. doi: 10.1097/MCG.0000000000001201 30882537 10.1097/MCG.0000000000001201

[98] Smyth A Judge C Wang X Pare G Rangarajan S Canavan M Chin SL Al-Hussain F Yusufali AM Elsayed A; INTERSTROKE Investigators. Renal impairment and risk of acute stroke: the INTERSTROKE study. Neuroepidemiology. 2021;55:206–215. doi: 10.1159/000515239 33951632 10.1159/000515239

[99] Boulanger M Poon MTC Wild SH Al-Shahi Salman R. Association between diabetes mellitus and the occurrence and outcome of intracerebral hemorrhage. Neurology. 2016;87:870–878. doi: 10.1212/WNL.0000000000003031 27473136 10.1212/WNL.0000000000003031 PMC5035156

[100] Seminog OO Goldacre MJ. Gout as a risk factor for myocardial infarction and stroke in England: evidence from record linkage studies. Rheumatology (Oxford). 2013;52:2251–2259. doi: 10.1093/rheumatology/ket293 24046469 10.1093/rheumatology/ket293

[101] Gutierrez J Albuquerque ALA Falzon L. HIV infection as vascular risk: a systematic review of the literature and meta-analysis. PLoS One. 2017;12:e0176686–e0176686. doi: 10.1371/journal.pone.0176686 28493892 10.1371/journal.pone.0176686 PMC5426615

[102] Behrouz R Topel CH Seifi A Birnbaum LA Brey RL Misra V Di Napoli M. Risk of intracerebral hemorrhage in HIV/AIDS: a systematic review and meta-analysis. J Neurovirol. 2016;22:634–640. doi: 10.1007/s13365-016-0439-2 27044037 10.1007/s13365-016-0439-2

[103] Ng CYH Tan BYQ Teo YN Teo YH Syn NLX Leow AST Ho JSY Chan MY Wong RCC Chai P. Myocardial infarction, stroke and cardiovascular mortality among migraine patients: a systematic review and meta-analysis. J Neurol. 2022;269:2346–2358. doi: 10.1007/s00415-021-10930-x 34997286 10.1007/s00415-021-10930-x

[104] Wiseman SJ Ralston SH Wardlaw JM. Cerebrovascular disease in Rheumatic diseases. Stroke. 2016;47:943–950. doi: 10.1161/STROKEAHA.115.012052 26917565 10.1161/STROKEAHA.115.012052

[105] Yazdany J Pooley N Langham J Nicholson L Langham S Embleton N Wang X Desta B Barut V Hammond E. Systemic lupus erythematosus; stroke and myocardial infarction risk: a systematic review and meta-analysis. RMD Open. 2020;6:e001247. doi: 10.1136/rmdopen-2020-001247 32900883 10.1136/rmdopen-2020-001247 PMC7722272

[106] Jin X Chen H Shi H Fu K Li J Tian L Teng W. Lipid levels and the risk of hemorrhagic stroke: a dose–response meta-analysis. Nutr Metab Cardiovasc Dis. 2021;31:23–35. doi: 10.1016/j.numecd.2020.10.014 33257190 10.1016/j.numecd.2020.10.014

[107] Qie R Liu L Zhang D Han M Wang B Zhao Y Liu D Guo C Li Q Zhou Q. Dose-response association between high-density lipoprotein cholesterol and stroke: a systematic review and meta-analysis of prospective cohort studies. Prev Chronic Dis. 2021;18:E45–E45. doi: 10.5888/pcd18.200278 33988499 10.5888/pcd18.200278 PMC8139481

[108] Zhou Z Liang Y Qu H Zhao M Guo F Zhao C Teng W. Plasma homocysteine concentrations and risk of intracerebral hemorrhage: a systematic review and meta-analysis. Sci Rep. 2018;8:2568–2568. doi: 10.1038/s41598-018-21019-3 29416106 10.1038/s41598-018-21019-3 PMC5803270

[109] Booth J Connelly L Lawrence M Chalmers C Joice S Becker C Dougall N. Evidence of perceived psychosocial stress as a risk factor for stroke in adults: a meta-analysis. BMC Neurol. 2015;15:233–233. doi: 10.1186/s12883-015-0456-4 26563170 10.1186/s12883-015-0456-4 PMC4643520

